# Whole genome characterization, and geographical distribution of M. tuberculosis in central region of Veracruz, Mexico

**DOI:** 10.1016/j.bjid.2022.102357

**Published:** 2022-05-06

**Authors:** Esdras Antonio Fernández-Morales, Gustavo Bermudez, Hilda Montero, Manuel Luzania-Valerio, Roberto Zenteno-Cuevas

**Affiliations:** a University of Veracruz, Health Sciences Institute, Health Sciences Master Program, Mexico; b University of Veracruz, Public Health Institute, Mexico; c University of Veracruz, Health Sciences Institute, Health Sciences Doctoral Program, Mexico; d Multidisciplinary Network of Tuberculosis Research, Mexico

**Keywords:** Tuberculosis, WGS, Geolocation

## Abstract

The purpose of this work was to perform by Whole Genomic Sequencing (WGS) a characterization of tuberculosis isolates circulating in the central region of Veracruz, Mexico, and to determine its geographical distribution. The genome of 25 clinical isolates of tuberculosis patients, recovered from central zone of Veracruz, Mexico, were sequenced and the information obtained was used to characterize lineage, prediction of drug resistance, identification of clonal complexes, and finally correlated with the geolocalization data. Isolates analyzed were included into seven L4 sublineages, most frequent was X3; X1 (4.1.1.3) in 35%. rpoBSer450Leu polymorphism was the most frequently found variant. Sublineage Haarlem (4.1.2) had the widest distribution, found in five municipalities. Of the of two clonal complexes found, the most abundant included eight isolates, with X3/X1 lineage, placed in two municipalities. Combination of WGS and geographic information system was very useful for the identification of sublineages, clonal complexes, and their geographical dispersion with important implications in the epidemiological surveillance and clinical control of TB.

Tuberculosis (TB) remains one of the most important infectious diseases in the world. According to the WHO global tuberculosis report 2020, almost 10 million people became ill, and 1.2 million deaths were due to TB.^1^

In the past decade, the standardization of whole genome sequencing (WGS) had an important impact on the characterization and understanding of TB, thanks to its higher resolution power to predict drug resistance, identify lineage, transmission routes, and geographic distribution.^2^^,^^3^ For these reasons, WGS has been used as a tool for the development of genomic surveillance programs in TB in developed countries. However, the use of WGS in low and middle-income settings, usually those with the highest burden of disease, has been scarce.^4^

In Mexico, TB has an incidence of 22 cases per 100,000 inhabitants, from which, 3% had resistance to at least one drug.^1^ Multidrug- (MDR-TB) and extensively drug resistant-tuberculosis (XDR-TB) have been increasing annually, placing Mexico among the five main TB contributors in the Americas.^5^ Unfortunately there are few studies related to the characterization of TB in Mexico, using WGS,^6^^,^^7^ thus limiting the evaluation of its effectiveness. Considering the above, this study aimed to use WGS to characterize the *M. tuberculosis* isolates circulating in the central region of the state of Veracruz, Mexico, and by use of geographic information system determine the geographic distribution of the lineages found.

Twenty-five sputum samples from patients with confirmed TB diagnosis from January 2018-July 2019 were randomly collected by the Mycobacteriosis Program from the Veracruz Ministry of Health in Mexico from 125 clinical cases detected in the period. This geographic area includes 25 municipalities, covers an area of 5,000 km^2^ and gives attention to more than one million inhabitants (Fig. 1).

**Fig. 1:**
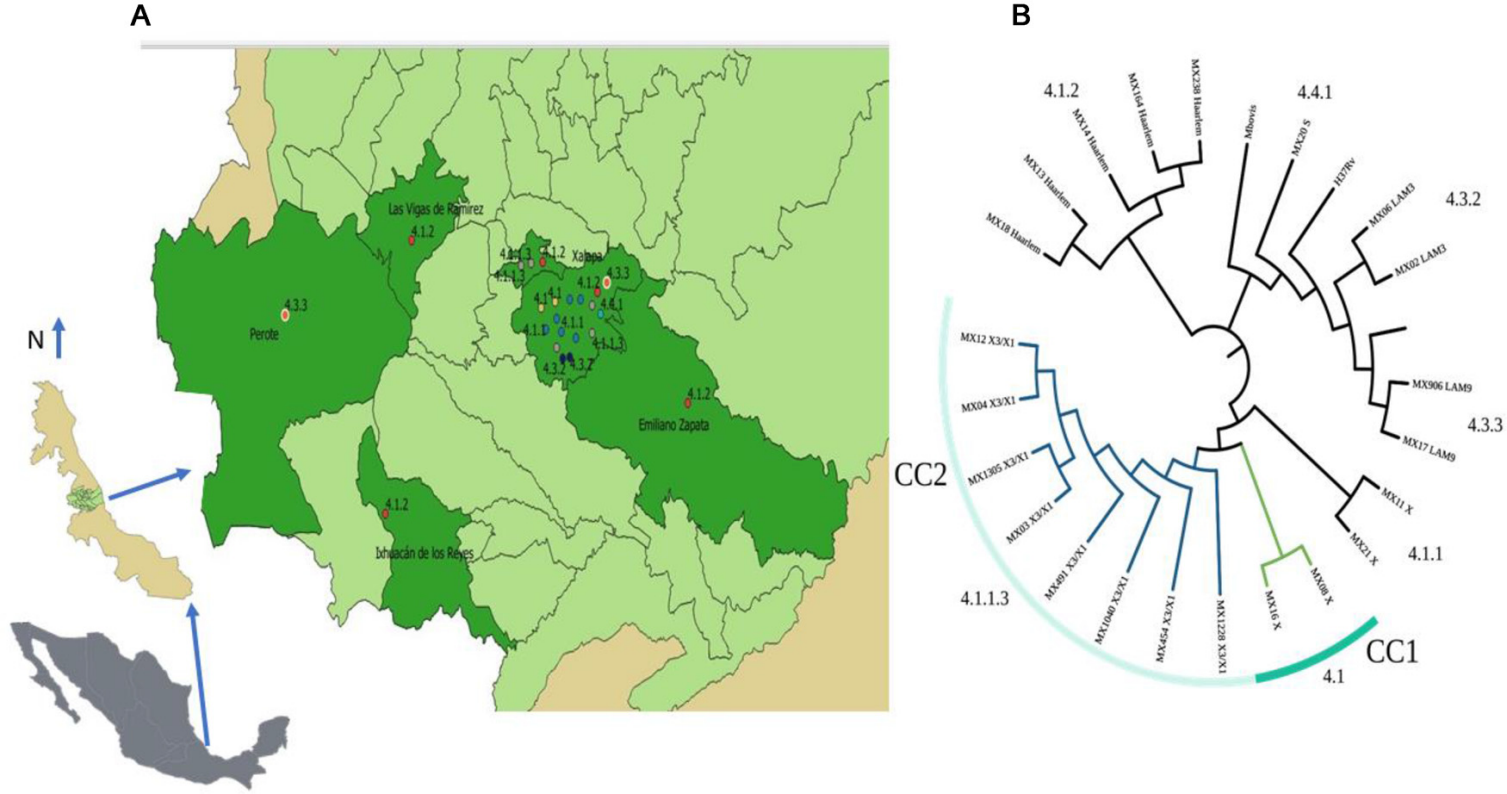
(A) Geographical distribution of *M. tuberculosis* lineages circulating in the center region of Veracruz, México. (B) Phylogenetic tree of isolates of circulating showing, lineages and clonal complexes found in the center region of Veracruz, Mexico.

Phenotypic drug sensitivity test (DST) against first-line drugs was performed by the staff of the Tuberculosis Program of the Health Secretary of the State of Veracruz using the fluorometric method (BACTEC, MGIT 960 Becton-Dickinson), according to standard conditions: isoniazid (H) > 0.1μg/mL, rifampin (R) 1.0 μg/mL, ethambutol (E) 5.0 μg/mL and streptomycin (S) 1.0 μg/mL. Pyrazinamide sensitivity was determined using a BACTEC MGIT 960 PZA kit (Becton Dickinson). Clinical, and epidemiological variables were abstracted from medical records.

*Mycobacterium tuberculosis* (MTB) strains were isolated in Lowenstein-Jensen media and DNA was extracted following CTAB method.^8^ Libraries were prepared according to Nextera XT protocol (Illumina, CA., USA), using 1 ng of DNA quantified by Qubit fluorometer (Invitrogen, CA, USA), library quality was determined using Bioanalyzer 2100 (Agilent Genomics), and sequenced using MiSeq (Illumina, CA., USA) in a 2 × 300 paired-end format.

Quality control of reads and trimmed was performed by using FastQC and Trimmomatic software, respectively. Reads were aligned using BWA,^9^ considering as reference, the genome of *M. tuberculosis* H37Rv (GenBank: NC_000962.3). Mapping, and variant calling was performed using a previously reported pipeline (http://tgu.ibv.csic.es/?page_id=1794).^10^^,^^11^ Variants in 20 reads, and at ≥ 90% of frequency within each isolate were called fixed-single nucleotide polymorphism (SNP), used to detect phylogenetic mutations. Variants in at least 10 reads at ≥10% to ≤90% frequency were called no fixed-SNPs and were used to detect antibiotic resistance. Analysis and selection of SNPs an INDELs related to antituberculosis drugs were done as describe by Madrazo-Moya et al.^12^

The data underlying the genome sequences presented in this study are available at ENA: https://www.ebi.ac.uk/ena. Accession number: PRJEB30933.

Strains were classified according to 62 phylogenetic variants associated with lineages as proposed by Coll et al.^13^ Phylogeny and genomic clusters were determined following recommendations by Jimenez-Ruano et al.^7^ and final tree visualization was done with Ito.^14^ Calculation of pairwise genetic distances between isolates considered a ≤ 12 SNPs threshold, as recommend Walker et al.^2^

Geographical distribution of the isolates (GIS) was carried out considering the geographic coordinates of the place of residence from the individuals bearing the isolates, and this was used to construct the spatial representation of the isolates using the geographic information system, QGIS (https://www.qgis.org/en/site/).

The results showed that individuals carrying isolates 88% (22) were male, mean age 43±13.2 years. The main comorbidity was type 2 diabetes mellitus at 32% (8), and 48% (12) reported a previous contact with a patient with TB in past year. Rifampicin resistance was observed in nine isolates (36%), followed by isoniazid in six (24%), pyrazinamide in three (12%), and five cases (20%) were MDR-TB.

According to the genomic resistance prediction, resistance to rifampicin was found in nine isolates (39%), followed by isoniazid in six (26%), pyrazinamide in seven (30%), ethambutol in one (4%), and six strains were MDR-TB (26%). Table 1 shows the most abundant variants identified, including one isolate with the variant *gyrAAsp94Gly,* that confers resistance to fluorquinolones (FQ) (Table 1). Five resistant isolates were confirmed by the WGS analysis. Nevertheless, four pyrazinamide sensitive isolates had the mutation *pncALeu120Pro*. One sensitive strain had mutations *rpoBSer450Leu,* and *katGSer315Thr*, another sensitive isolate carried the mutation *embBMet306Ile*, and one MDR-TB isolate was absent of any mutation (Table 1).

**Table 1:** Genotypic (r) vs phenotypic (R) drug sensitivity profile of drug resistant isolates circulating in center region of Veracruz, Mexico.*

Isolate (Lineage)	Phenotypic (R) vs genotypic (r) resistance				Isoniazid (H) *katG*	Rifampicin (R) *rpoB*			Ethambutol (E) embB	Pyrazinamide (Z) *pncA*	Fluoroquinolone (FQ) *gyrA*
	H	R	E	Z	2155168 Ser15Thr agc/acc	761108 Met 434 Ile atg/att	761109 Asp435Tyr gac/tac	761155 Ser450Leu tcg/ttg	4247431 Met 306 Ile atg/att	2288883 Leu120Pro ctg/ccg	7582 Asp94Gly gac/ggc
**No clustered drug resistant isolates**											
MX06 (LAM3; 4.3.2)		R/r						+			
MX164 (Haarlem; 4.1.2)		R/r						+			
MX238 (Haarlem; 4.1.2)		R/r				+	+				
MX906 (LAM9; 4.3.3)	R/-	R/-									
**Drug resistant solates placed in CC2 (X3/X1; 4.1.1.3)**											
MX03 (X3/X1)				-/r						+	
MX12 (X3/X1)	-/r	-/r		-/r	+			+		+	
MX491 (X3/X1)	*R/r*	*R/r*		*-/r*	+			+		+	
MX454 (X3/X1)	R/r	R/r		-/r	+			+		+	
MX1040 (X3/X1)	*R/r*	*R/r*	*-/r*	*R/r*	+			+	+	+	
MX1228 (X3/X1)	R/r	R/r		R/r	+			+		+	
MX1305 (X3/X1)	R/r	R/r		R/r	+			+		+	+

⁎ Twelve isolates were sensitive by drug resistance profile and confirmed by WGS.

Twenty-three isolates were included in seven L4 sublineages: 4.1 (X) and 4.1.1 (X) with two isolates, 4.1.1.4 (X3/X1) with eight, 4.1.2 (Haarlem) with five, 4.3.2 (LAM3) with two, 4.3.3 (LAM9) with three and 4.4.1 (S) with one strain only (Fig. 1b). The most frequent sublineage was X3;X1 (4.1.1.3) with eight isolates (35%), followed by 4.1.2 (Haarlem) with five. Only six municipalities from the central region of Veracruz contributed with TB isolates (Fig. 1a). Xalapa had the highest number of isolates with sixteen (70%), including all sublineages and four exclusive sublineages: 4.1 (X), 4.1.1 (X), 4.3.2 (LAM3), and 4.4.1 (S). The sublineage 4.1.2 (Haarlem), was the most widely distributed and observed in five municipalities.

Two clonal complexes (CC) were identified (Fig. 1a). The CC1 included two sensitive isolates with X sublineage (4.1.1) and placed in Xalapa, and the CC2 included eight isolates with lineage X3/X1 (4.1.1.3), with a circulation restricted to Xalapa and Banderilla (Fig. 1b). Five isolates in this group shared the same drug resistance and the polymorphisms associated, two strains showed different drug patterns, and one was sensitive.

Although this study involved a small number of patients the sociodemographic, and clinical variables agree with previous reports.^15^ Comparative analysis between phenotypic drug resistance test and WGS prediction confirmed the diagnostic in almost all isolates. However, some inconsistencies were observed: four pyrazinamide sensitive isolates had the mutation *pncALeu120Pro*, one sensitive had three highly confident mutations conferring resistance to H, R, and Z, and one MDR-TB isolate had no mutation that could explain such condition. The discrepancy specifically related to pyrazinamide could be explained by the fact that diagnostic test against this drug frequently shows discrepancies in the cutoff value considered for positivity, and the remaining differences could be explained by the algorithms used for SNPs detection, the variant catalogs used, and the cut-off values used for the identification of the variants related with drug resistance.^16^^,^^17^

The isolates analyzed were placed in seven L4 sublineages, which have been previously described as predominant in Mexico,^6^^,^^7^ and Latin America, and explained by historical events related with European colonization, and further migration of population in the continent.^18^^,^^19^

Eight isolates included in the CC2 sharing the X3/X1 sublineage (4.1.1.3) were observed in the two adjacent municipalities of Xalapa and Banderilla, and according to the drug resistant profile, seven of these strains had drug resistance. The sublineage 4.1.1.3, previously described in Mexico, is strongly associated with multidrug resistance,^7^ confirming the wide distribution of this sublineage in the country and the strong association with drug resistance. The municipality of Xalapa contained the highest number of cases, sublineages, and CCs, and most of them were placed in marginal colonies. This concentration could be explained by the fact that is the capital city of the state and have an important number of inhabitants living in poverty, marginalization, overcrowding, and precarious urban conditions.

To our knowledge, this is the first characterization of TB by WGS and the combined analysis with the geographic location and distribution of specific sublineages and CCs circulating in Mexico. These results confirm the diversity of sublineages circulating and the usefulness of GIS to generate a better definition of the epidemiological behavior of TB, similar to that described in China and Myanmar.^20^^,^^21^

The fact that 43% of the isolates were grouped into two clonal complexes, evidence the high transmission level of some TB clones in the population, and the need to strengthen the contact studies in active cases to avoid major dispersion of these strains, and also confirm the need to include WGS analysis in epidemiological surveillance.

Despite the main limitation of the study being the low number of isolates analyzed, it is possible to conclude that combination of WGS and GIS have a deep impact in the characterization of the tuberculosis isolates circulating improving the description of distribution and dispersion of sublineages and clonal complexes. This combination will have important implications in terms of epidemiology, surveillance, and control of TB.

## Statement of ethics

All aspects related with this research were approved by the Ethics Committee of the Public Health Institute of the Universidad Veracruzana (CEI-ISP-R18/2020). All subjects gave their written informed consent and ll protocols were performed in accordance with the national guidelines and regulations.

## Funding

EA F-M was funded by CONACyT, fellow number 850447. R Z-C was partially funded by CONACYT No. A1-22956 and ICOP-COOPB20344.

## Authors’ contributions

EAF-M, conceptualization, Data curation, Formal analysis, Investigation, Methodology, Software, Supervision, Validation, writing original draft, Writing review & editing. GB, Data curation, Formal analysis, Methodology, Validation, Visualization. MLV, Methodology, Validation, Visualization, Writing original draft. HM, Data curation, Visualization, writing original draft, Writing review & editing. RZ-C, conceptualization, Formal analysis, Funding acquisition, Investigation, Methodology, Project administration, Supervision, Validation, writing original draft, Writing review & editing.

## Conflicts of interest

The authors declare no conflicts of interest.

## Data Availability

The data underlying the genome sequences presented in this study are available at ENA: https://www.ebi.ac.uk/ena. Accession number: PRJEB30933.
